# Improving photoprotection in adults with xeroderma pigmentosum: personalisation and tailoring in the ‘XPAND’ intervention

**DOI:** 10.1080/21642850.2020.1840379

**Published:** 2020-11-07

**Authors:** Kirby Sainsbury, Jessica Walburn, Lesley Foster, Myfanwy Morgan, Robert Sarkany, John Weinman, Vera Araujo-Soares

**Affiliations:** a Population Health Sciences Institute, Faculty of Medical Sciences, Newcastle University, Newcastle Upon Tyne, UK; b School of Cancer & Pharmaceutical Sciences, King’s College London, London, UK; c XP NCG Service, Guy’s and St. Thomas’ Hospital NHS Foundation Trust, London, UK

**Keywords:** Individualised, behaviour change intervention, rare disease, intervention development, UVR protection

## Abstract

**Background:**

Individualised behaviour change interventions can result in greater effects than one-size-fits-all approaches. Factors linked to success include dynamic (vs. static) tailoring, and tailoring on behaviour, multiple theoretical variables, and participant characteristics. XP is a very rare (∼100 UK patients) genetic disease, involving an inability to repair ultraviolet radiation (UVR)-induced damage, resulting in skin cancers and eye damage from an early age, and mean life expectancy of 32-years. Management involves rigorous UVR photoprotection, which is often inadequate, and no interventions have been published. UK-based care is personalised and delivered by a multidisciplinary team at the National XP Service in London. Following an intensive, mixed-methods formative phase with patients diagnosed with XP (n-of-1, qualitative interviews, objective UVR measurement, cross-sectional survey) and relevant stakeholder consultation (clinical and patient/public teams), the ‘XPAND’ intervention was developed. This paper describes the comprehensive and novel tailoring and personalisation processes used to deliver the intervention.

**Methods:**

XPAND consists of core and personalised modules targeting cue-based (time of day, weather, symptoms), belief-based (motivation, priority), self-regulatory (effort, barriers, planning), and emotional (stress, self-consciousness, mental exhaustion) factors, social support, disclosure, habit, and willingness, using appropriately-matched BCTs. A-priori, phase I data and a baseline profiling questionnaire (data sources) were used to allocate modules to participants (‘personalisation’) and to adapt module content (‘tailoring’). Iterative decisions about delivery were based on patient response to feedback, identification of additional barriers (e.g. reasons for varying protection across contexts), and emergence of new barriers as improvements in protection were attempted or achieved (e.g. appearance concerns).

**Conclusions:**

Dynamic multi-level personalisation and tailoring based on mixed-methods in XPAND allowed for insights and decision-making not possible with cross-sectional quantitative or qualitative methods alone. Data collection and allocation/adaptation methods may be of use in other rare conditions where small patient numbers mean that within-participant, individual-level delivery is well-suited and feasible.

## Introduction

Movement from ‘one-size-fits-all’ treatments to personalised health care has recently been observed across medical and psychological fields. For example, precision medicine offers the potential to identify underlying disease and genetic mechanisms, which, combined with the capturing of large-scale data on health and habits (e.g. food and alcohol consumption, physical activity), can be used to predict individual risk and facilitate early and accurate diagnosis (National Research Council, 2011). Consequently, preventive measures, drug and other treatments (e.g. health behaviour changes) can be matched to patient profiles, including the presence of known indicators of likely responsiveness to treatment, to ensure the greatest likelihood of success (Graham, 2016). Effective clinicians across fields have long engaged in the personalisation of treatments for their patients; for example, in the context of individual psychological consultations (with a clinical or health psychologist), treatment strategies are selected and matched to the unique formulation of each patient’s presenting problem and identified or hypothesised maintaining factors (Johnstone & Dallos, 2014; Nikcevic, Kuczmierczyk, & Bruch, 2006). Given the number of people in need (e.g. based on statistics for engagement in unhealthy lifestyle behaviours), however, the reach and feasibility of achieving wide-scale population change with such interventions is typically low. Unsurprisingly, the application of personalised approaches to larger-scale lifestyle problems has lagged behind clinical practice, with standardised interventions still dominating the literature and public health spheres. The lack of opportunity for real-world implementation of effective tailored interventions represents another challenge (Noar, Grant Harrington, Van Stee, & Shemanski Aldrich, 2011), although improved technologies for data recording and dissemination are increasingly allowing for wider-scale application.

The known complexity of human behaviour combined with the modest effectiveness of interventions and heterogeneity of treatment effects (observed in most meta-analyses of behaviour change interventions), strongly suggest that greater precision in selecting psychological and behaviour change interventions for individuals is needed to really make progress in the management and prevention of problems that stem, at least in part, from engagement in unhealthy behaviours and/or inadequate performance of healthy behaviours. The purpose of this paper is to describe the ways in which an intervention designed to improve adherence to photoprotection recommendations in patients with the rare and life-limiting disease, xeroderma pigmentosum (XP), was personalised and tailored to individuals. Before detailing the condition or developed intervention, we review the literature on personalisation and tailoring, with a focus on the findings most pertinent to a rare disease involving complex self-management and for which routine clinical care already involves an individualised and face-to-face approach.

### The case for tailoring interventions and how tailoring works

Tailoring has been broadly defined as ‘any combination of strategies and information intended to reach one specific person, based on characteristics that are unique to that person, related to the outcome of interest, and derived from an individual assessment’ (Kreuter, Strecher, & Glassman, 1999, p. 277). ‘Segmentation’ (i.e. the dividing of the target audience into increasingly narrow and homogenous groups) and ‘customisation’ (i.e. the delivery of messages that are matched to relevant individual characteristics), as well as personalisation, feedback, and content-matching all provide means to the achievement of tailoring goals (Hawkins, Kreuter, Resnicow, Fishbein, & Dijkstra, 2008). Despite these distinctions, the terms ‘tailoring’ and ‘personalisation’ are often used interchangeably in the literature and as umbrella terms under which various more-specific strategies fall. Latterly, ongoing work to establish definitional consensus (personal communication, Dr Marta Marques, January 29, 2018) has suggested that, in the context of behaviour change, ‘tailoring’ can be defined as the process of modifying or adapting the standard content of an intervention to suit the personal needs, characteristics, or preferences of an individual. ‘Personalisation’ refers to decisions regarding whether, in which order, how frequently, and by what means (e.g. mode of delivery) particular pieces of standardised information and strategies from a wider content pool should be delivered. After the allocation of content to individuals (‘personalisation’ by the above distinction), further tailoring may or not be undertaken; for example, adapting standard content of the pre-selected modules to the individual. Both processes require an in-depth knowledge of the relevant user attributes (e.g. correlates of behaviour, motivation or confidence to change, preferences), derived from standardised individual assessment, so that interventions can be most-appropriately matched and delivered. For simplicity and consistency with prior literature, we will use the term ‘tailored’ throughout the introduction to refer to any attempt to increase the personal relevance of an intervention for an individual, although later adopt the more nuanced terms, as defined above, to describe the target intervention.

Conceptually, tailored interventions should be preferable and more effective than standardised, non-tailored interventions in achieving desired outcomes (e.g. behaviour change), as specified in the elaboration likelihood model (Petty & Cacippo, 1986). The hypothesised mechanisms underlying this assertion are that information perceived as personally relevant encourages increased attention, engagement, processing (effortful and emotional), and acceptance of that information, incites self-referential thinking and motivation, and acts on other known determinants of change (e.g. self-efficacy), which are favourable, although not necessarily sufficient, conditions for behaviour change (Hawkins et al., 2008). Meta-analyses and reviews have supported the superiority (compared to non-tailored) and/or effectiveness (compared to no-treatment control conditions) of tailored interventions across a range of health behaviours, and spanning computer-tailored (i.e. where computer algorithms are used to make tailoring decisions), and web, phone, print, and face-to-face delivery (a comprehensive review is beyond the scope of this paper; see Krebs, Prochaska, & Rossi, 2010; Lustria et al., 2013; Noar, Benac, & Harris, 2007; Noar et al., 2011; Wanyonyi, Themessl-Huber, Humphris, & Freeman, 2011).

The processes of tailoring behaviour change interventions vary across studies. The ‘black box of tailoring’ refers to how tailoring works and the specific aspects that lead to increased intervention effectiveness. Moderator analyses reported in the previously mentioned meta-analyses (all of which combined interventions for different behaviours to avoid conflating behaviour-specific and tailoring effects) have shed light on some of these factors. First, increased intervention contact points were associated with larger effects in computer-tailored interventions (Krebs et al., 2010; Noar et al., 2007). The number of assessments on which feedback and tailoring was based also impacted effect size (Krebs et al., 2010). That is, ‘static’ tailoring, whereby a one-off baseline assessment occurs, was found to result in weaker effects than interventions that employed ‘dynamic’ tailoring, where feedback is updated throughout the intervention based on changes identified via repeat assessment. Statically tailored interventions with multiple contact points had larger effects than those with a single contact point, although only the dynamically tailored interventions resulted in longer-term maintenance of effects (Krebs et al., 2010). Considering the hypothesised mechanisms via which tailoring improves effectiveness (i.e. perceived relevance, which should remain high when feedback is responsive to change, and conversely, may decrease if information continues to be selected based on baseline cognitive and/or behavioural patterns that have since changed in response to the intervention) and typically observed dose–response relationships, these findings may not be entirely surprising, but they do suggest ways that effects in future tailored interventions can be maximised and maintained.

In contrast, a meta-analysis of web-delivered interventions did not find an advantage of dynamic over static tailoring (Lustria et al., 2013). This was, however, likely attributable to the observation that most dynamically-tailored interventions were conducted in chronic conditions (i.e. involving complex self-management vs. briefer preventive interventions with single behaviour change targets) with high-risk or patient groups (vs. general population) and involved longer-term follow-up (whereby deterioration of effects over time is common vs. short-term studies, which likely capture effects at their prime), all of which were associated with weaker effects in moderator analyses (Lustria et al., 2013). This finding may also indicate that web-based delivery in complex conditions is not optimal due to the more ambitious nature of sustained engagement and behaviour change needed to achieve improvements. No difference in effect was seen according to the level of involvement from an expert within the web-delivered interventions (self-guided vs. expert-guided; Lustria et al., 2013).

Effects also vary depending on the number and combination of variables used to tailor. In a meta-analysis of computer-tailored, print-delivered interventions, tailoring was based on either behaviour only, theoretical constructs only, theory plus demographics, theory plus behaviour, or theory plus demographics *and* behaviour (Noar et al., 2007). The greatest effects were observed in interventions that tailored based on all three factors; using theory and behaviour (without demographics) resulted in a smaller although statistically-equivalent pooled effect, suggesting that tailoring using the combination of behaviour and its drivers is ideal, with relevant demographic tailoring having the potential to add further value. Those that used behaviour only (resulting in short and simple normative feedback) had the weakest effects, followed by theory only, although some of this effect may, again, be expected simply based on the reduced dose and complexity of feedback derived from single sources. Consistent with this explanation, effects increased when 4–5 theoretical constructs were used to tailor information compared to fewer (0-3; Noar et al., 2007). Similarly, in a meta-analysis of tailored interventions involving face-to-face delivery in primary care settings, stronger effects were observed when tailored messages were combined with either brief advice or repeated follow-ups (i.e. increased dose; Wanyonyi et al., 2011).

Finally, although increased effects were seen in interventions that tailored on the basis of attitudes, self-efficacy, stage of change, social support, and processes of change, tailoring to perceived susceptibility resulted in weaker effects (Noar et al., 2007). This is consistent with evidence that increasing the salience of disease-related threat is only effective under certain conditions (i.e. when self-efficacy is also successfully targeted; Peters, Ruiter, & Kok, 2013), and points to the importance of carefully selecting the theories on which interventions and tailoring are based. The most commonly-used theories for tailoring were the transtheoretical stages of change model and social cognitive theory (Noar et al., 2007; Wanyonyi et al., 2011), whereas the face-to-face interventions also frequently used motivational interviewing (Wanyonyi et al., 2011).

### Personalised care planning and shared decision-making

While not strictly classified as ‘tailored’ due to the absence of a standardised assessment and the adaptation of standardised content to the individual, other relevant classes of face-to-face interventions used in clinical care include ‘personalised care planning’ and ‘shared decision-making’. The former is a highly personalised approach in which patients are encouraged to become involved in goal setting and action planning with a healthcare professional to support behaviour change and improve self-management, rather than the healthcare professional being the sole decision-maker (Coulter et al., 2015). The latter was defined in a consensus conference as ‘a conversation between the clinician and patient in which they figure out together what to do to address the patient’s situation’ (Kunneman, Montori, Castaneda-Guarderas, & Hess, 2016, p. 1320) and which, more specifically, involves establishing the diagnosis, clarifying the available treatment choices, discussing the harms and benefits of each and fit with patient situation, and making a decision (Hargraves, LeBlanc, Shah, & Montori, 2016).

A Cochrane review of personalised care planning interventions for people with long-term conditions (e.g. diabetes) found a small but positive effect on outcomes (physical and psychological health, self-management capabilities) compared to usual care (Coulter et al., 2015). Consistent with the advantages of increased contact points/dose and dynamic tailoring (inherent in personalised care planning), greater effects were observed when interventions were more comprehensive, intensive, and better integrated into routine care (Coulter et al., 2015). A systematic review of shared decision-making interventions found that while there was currently a lack of evidence for impacts on behavioural or health outcomes, a positive effect on cognitive–affective outcomes was often seen (Shay & Lafata, 2015), suggesting that, even in the absence of improved health, patient satisfaction and acceptability, akin to several engagement-based mechanisms via which tailored interventions can exert their effects, are likely to be maximised using a shared approach. A meta-analysis also found that shared decision-making interventions improved outcomes for disadvantaged patient groups (e.g. those with lower literacy), including positive effects on knowledge, participation, and decision self-efficacy (Durand et al., 2014), further indicating their potential over one-size-fits-all interventions. Combining these avenues of research, the amalgamation of personalised face-to-face contact within routine care and message tailoring (i.e. the adaptation of standardised content to individual characteristics) may offer potential for improved outcomes in complex conditions where web-delivered interventions have been more limited.

### The disease: xeroderma pigmentosum (XP)

XP is a very rare disease, affecting 2.3 per million lives births (Lehmann, McGibbon, & Stefanini, 2011), with ∼100 known cases in the UK. Affected individuals have a defect in the genetic pathway responsible for repairing ultraviolet radiation (UVR)-induced DNA damage, so exposure across the lifespan is cumulative (Fassihi, 2013). Clinical manifestations can include an abnormally severe sunburn reaction after minimal UVR exposure, neurological difficulties (e.g. balance, cognition, hearing), and an increased risk of melanoma (2000-fold) and non-melanoma skin cancer (10,000-fold) prior to the age of 20 years, and eye disease including corneal and conjunctival cancers (Bradford et al., 2011). The only way to protect against the cancers and have a longer lifespan is rigorous photoprotection against daylight, which includes adapting activities to minimise the duration of time spent outdoors, particularly in higher-risk times (e.g. 11am-3pm) and, when outdoors, wearing protective clothing (e.g. face visor, hat, sunglasses) and frequently applying broad-spectrum SPF50+ sunscreen (Tamura, DiGiovanna, Khan, & Kraemer, 2014). Prior to 2015, no research had been conducted on the behavioural or psychosocial characteristics of people diagnosed with XP, and no attempts at intervention development had been made.

### The case for intervention tailoring in XP

There is good evidence to suggest that tailoring can work, and data suggesting how to best implement tailoring strategies for effectiveness. The nature of the target behaviour and population, and feasibility, are also relevant considerations when determining whether, and to what degree/depth, to embark on the development of a tailored intervention. In adherence research, there is an emerging view that no single theory can encompass the complexity of factors associated with adherence behaviour (Easthall & Barnett, 2017; Holmes, Hughes, & Morisson, 2014). By extrapolation, no single set of intervention strategies could be expected to achieve change for all affected individuals, with more individualised approaches being recommended (Easthall & Barnett, 2017). Even more specifically, in the context of adherence to a rare disease, where patient numbers are small and there is considerable heterogeneity in behaviour and its drivers, the use of tailored approaches may represent the only appropriate option. Where the trade-off between reach and efficacy is a difficult balance to strike in interventions for more common conditions and behaviours, tailoring is very well-suited and highly feasible in a rare disease (Sainsbury, Walburn, Araujo-Soares, & Weinman, 2018a).

In 2015, the National Institute of Health Research (NIHR, UK) funded the ‘XP project’, which has the overall goal of developing and testing, in a randomised controlled trial, a toolbox of interventions to improve adherence to photoprotection recommendations, and that could eventually be integrated into routine clinical care (Walburn et al., 2019b). Initially, this involved the conduct of a series of in-depth studies using mixed-methods (e.g. qualitative interviews, n-of-1, international cross-sectional survey, objective UVR measurement) to determine the extent to which patients with XP performed the recommended photoprotective behaviours, and suggest modifiable drivers of protection (e.g. motivation, barriers and facilitators; phase I) that could be targeted to achieve change in the planned intervention (phase II). Outcomes of this formative research (Anderson, Walburn, & Morgan, 2017; Anderson, Walburn, & Morgan, 2019; Morgan, Anderson, Walburn, Weinman, & Sarkany, 2019; Sainsbury et al., 2018b; Walburn et al., 2019a; Walburn, Anderson, & Morgan, 2019c) and the development of the intervention content based on phase I findings (Walburn et al., 2020) are reported separately. Briefly, this phase of research resulted in the establishment of 17 evidence statements around which the intervention would be designed and confirmed that, not only did adherence to photoprotection recommendations vary widely, so did the number and pattern of correlates of behaviour, lending further support to the a-priori decision that the planned intervention should be tailored.

The UK National Health Service (NHS) XP clinical service, based at St Thomas’ Hospital, London (NHS England, 2018a), is a ‘highly specialised service’ (defined as an NHS service that provides specialised care to a small number of patients), informed by the Rare Diseases Advisory Group and the UK Strategy for Rare Diseases (which includes a focus on promoting research; NHS England, 2018b). It is comprised of a multi-disciplinary team of Dermatologists, Dermatological Surgeons, Specialist Nurses, Clinical Geneticists, Ophthalmologists, Neurologists, and Neuropsychologists, with strong links to the XP patient support group. Care is delivered via regular XP clinics where patients attend for a full day and are seen by a range of healthcare professionals, as indicated by their current health and management needs. Thus, routine care is already designed using individualised protocols, although clinical observations of inadequate adherence suggested that additional and targeted behaviour change support would be of benefit in achieving optimal outcomes for patients. Greater degrees of tailoring, and the associated increase in cost and effort, are said to be ‘worth it’ if there is a sufficient variability in behaviour and its determinants and a feasible mechanism for data collection and the delivery of customised health information (Hawkins et al., 2008). In XP, both these conditions are met: the phase I studies showed considerable variability, and the NIHR-funded XP project and well-established NHS clinical service, including care coordination by the Clinical Nurse Specialists, provide means for delivery, both within the trial and looking to future integration into clinical care pathways.

Following calls to improve the reporting of the ways in which tailoring decisions are made and implemented (e.g. Noar et al., 2011; Wanyonyi et al., 2011), the goal of this paper is to describe how the developed intervention – ‘XPAND’ – was tailored and personalised to individual participants, and how such decisions were informed by previous evidence for the components of tailoring linked to success.

## Methods

### The XPAND intervention

For a full description of the development and content of the XPAND intervention, please refer to our companion paper (Walburn et al., 2020). Briefly, XPAND involved 7, one-to-one sessions delivered in person (sessions 1 and 6) or via Skype (sessions 2–5 and 7) by a trained facilitator. It was accompanied by the provision of a purpose-designed magazine, session worksheets, a video illustrating correct application of sunscreen and the process of habit formation, and a personalised feedback sheet detailing their protection (e.g. time spent outdoors, protection used, proportion of time protected by sunscreen), as collected in phase I. It contained both core intervention components (e.g. goal setting, action and coping planning, habit formation), delivered to all participants, and personalised modules, selected using the processes described below. Relevant behaviour change techniques (BCTs; Michie et al., 2013) were selected and delivered alongside strategies drawn from Acceptance and Commitment Therapy (ACT; Harris, 2013) and Cognitive Behaviour Therapy (CBT; Clarke, Thompson, Jenkinson, Rumsey, & Newell, 2013).

The seven personalised modules were developed to target: (1) stress and its interaction with protection (whereby stress – and low mood – could conceivably be the cause *or* consequence of the chosen level of protection; included strategies to manage stress ‘in the moment’ and prevent stress and resource depletion over time); (2) mood and its interaction with protection (included behavioural activation strategies to boost mood, and cognitive defusion to manage unhelpful thinking and prompt values engagement even in the presence of unhelpful thoughts); (3) values (extension of the session 1 exploration of personal reasons for protection to explicitly consider values-based motivation; included specific ACT strategies for how to use values as motivators; intended predominantly for participants identified as ‘resistant’ to protection and the XP identity in the phase I qualitative interviews); (4) willingness (framed as the ‘bridge’ between motivation and improved protection when barriers cannot be fully removed; included specific ACT strategies such as the willingness and action plan); (5) appearance concerns (included CBT-based strategies to manage unwanted attention from strangers and feelings of self-consciousness, social engagement skills to boost confidence, and references to willingness for when some level of discomfort remains despite use of other strategies); (6) social support (included focus on both practical and emotional support, toxic and unhelpful support, and a sub-module on disclosure for use if non-disclosure of XP was the reason for insufficient support being available); and (7) necessity (divided into seven sub-modules targeting different necessity beliefs that were relevant to inadequate protection; see Table 2; all sub-modules included strategies to elicit and amplify ‘necessity talk’ and look for behavioural exceptions in the presence of doubts).

Delivery of the intervention was consistent with a Motivational Interviewing (MI) approach, including the use of collaboration, acceptance, compassion, and evocation (Rollnick & Miller, 1995). The combination of ACT and MI principles informed the ‘spirit of XPAND’ (akin to the ‘spirit of MI’), whereby facilitators communicated with participants in a non-judgmental, curious, and collaborative manner; emphasising the natural course of behaviour change (i.e. non-linear); modelling acceptance and compassion, as well as problem-solving in the face of challenges; eliciting and reinforcing ‘change talk’, including a focus on positive emotional consequences of better protection; and striking a balance between the two main approaches to improve photoprotection: (1) reducing barriers or developing strategies to better manage them, and (2) developing internal, value-based motivations and willingness to protect, even in the presence of barriers. Using this approach, a key goal of XPAND was to achieve improvements in photoprotection, such that habitual good protection became the backdrop on which engagement with values and other life priorities occurred, rather than reducing available time and resources for this essential task. The strong focus on values to guide protection decisions also meant that the potential emotional toll of good photoprotection (identified in the phase I research) was managed.

### Personalisation and tailoring in XPAND

**‘**Personalisation’ refers to the allocation of standardised content to each participant in an intervention, based on an assessment of their individual characteristics, while ‘tailoring’ refers to the adaptation of that content to the individual (personal communication, Dr Marta Marques, January 29, 2018). In the XPAND intervention, both personalisation (allocation) and tailoring (adaptation) were used to supplement and enhance the delivery of the core intervention content that was delivered to all participants, to maximise the ‘goodness of fit’ between individual needs and treatment provision (Kreuter & Wray, 2003). Drawing on literature suggesting the components of tailoring linked to effectiveness and informed by stakeholder (XP clinical team and patient and public involvement (PPI) panel) involvement to specify the requirements that the intervention needed to fulfil (e.g. eventual delivery in routine care, engaging and attractive materials, focused on long-term maintenance), the following strategies were chosen for implementation in XPAND:

- Multiple contact points (although this was standardised across participants)
- Dynamic tailoring
- Tailoring on the basis of behaviour *and* multiple theoretical variables
- Additional tailoring to patient characteristics (e.g. language/literacy levels and preference for cognitive vs. behavioural strategies within modules)
- Predominantly face-to-face (in person and Skype) delivery (standardised across participants)
- Purpose-designed worksheets and magazine for use in and between sessions
- Text messages between modules to prompt goal completion and support habit formation, personalised based on individual drivers of behaviour and delivered content

While the chronic and high-risk nature of XP and complexity of self-management cannot be altered (all of which were moderators of tailored intervention effectiveness), the involvement of the clinical and PPI teams in the intervention development process, and integration of components of shared decision-making and personalised care planning protocols (e.g. collaborative and iterative decision-making about intervention content and the setting of behavioural goals and action plans), meant that potential threats to efficacy were minimised.

### Data sources

Intervention decisions were informed by a range of data sources at different points in the intervention delivery process, and used to tailor at multiple levels (i.e. behaviour, drivers of behaviour, direction of relationship/specifics of each driver). Table 1 summarises the different data sources and how they were used, while Table 2 provides a more detailed description of the mapping of those data sources to the personalised intervention modules (described previously) and the strategies used to tailor the standardised content within each module. Phase I data included that from a daily UVR diary and n-of-1 study (both completed over a 7-week period), qualitative interviews, and an international cross-sectional survey. The methods and results from these studies are described only briefly here; please refer to the published papers for full details.

- *The UVR diary study* involved completion of a time-use diary and allowed participants to record information about the time spent outside and photoprotection (clothing combinations and sunscreen application) they used when outside (see Sainsbury et al., 2018b). All possible behaviour combinations (e.g. hat only; hat + glasses; hat + glasses + hoodie) were ranked by the clinical team to indicate the level of protection provided and associated level of risk, relative to the ‘ideal’ (i.e. use of a face visor, which blocks 99% of UVR). The resultant categories formed the Daily Protection Scale (DPS), which ranges from ‘very poor’ to ‘excellent’ protection. Results showed that protection was highly variable and inadequate for most participants; only 4/20 ever used a visor (8-86% of all outdoor time; ‘excellent’ protection); 13 participants were using none or ‘very poor’ protection for at least 20% of outdoor time; only 10 participants were using at least ‘good’ protection (i.e. ‘good’, ‘very good’, or ‘excellent’) at least 50% of the time; sunscreen was only used for a median of 57% of outdoor time. The information obtained from the UVR diary and summarised using the DPS was the basis for behavioural feedback given to each participant in session 1 (described later).
- *The n-of-1 study* used behavioural data from the UVR diary (as described above) and additionally involved completion of a 22-item mobile phone survey each evening, assessing a range of psychosocial constructs (see Sainsbury et al., 2018b). Data was analysed, per person, using dynamic logistic regression (outcome: DPS category) and Spearman’s correlations (outcome: self-reported photoprotection, 0-100), depending on the level of observed variability in behaviour and predictors. Across these different analyses, correlates of photoprotection included environmental factors (high risk vs. lower risk time of day, weekday vs. weekend, social support, perceived risk, how sunny it was, UVR-related symptoms); belief-based (motivation, confidence, importance), self-regulatory (effort, planning, barriers), and emotional factors (related to XP and protection: negative thoughts, missing out, stress, self-consciousness; general/not related to XP: mental exhaustion, mood, quality of life, energy level), although the direction of relationships differed across participants. Overall, self-reported protection (the outcome that was available for the most number of participants) was related to at least one environmental factor for 14/18 participants, at least one self-regulatory factor for 16/18, at least one belief-based factor for 10/18, and at least one emotional factor for 15/18 participants (XP-related: n=11; general: n=12).
- *The qualitative interview* framework analysis revealed three distinct groups of participants who differed in their level of protection and adjustment to the diagnosis and required management (see Morgan et al., 2019). The ‘resistant’ group (n=11) had limited photoprotection and haphazard routines; they resisted the identity of somebody with a chronic condition and instead prioritised living a ‘normal’ life above photoprotection; they often doubted the need for protection and/or its effectiveness; and showed reluctance to disclose their condition. The ‘integrated’ group (n=10) accepted XP and had managed to integrate photoprotection into their everyday lives, though often with room for improvement; most of their practices had become habitual; management was not described as causing a major practical or emotional burden. The ‘dominated’ group (n=4) had a high level of photoprotection but the time and planning involved in management dominated their lives and this often came with a cost to emotional wellbeing. Given that the aim of XPAND was to improve protection, this group were not eligible for participation, although the observation of an emotional toll to good protection was a key consideration in the intervention design process. Additional themes relevant to photoprotection and illness adjustment were the experience and management of stigma (see Anderson et al., 2017) and the importance of social support (see Walburn et al., 2019c).
- *The cross-sectional survey* (see Walburn et al., 2019a) involved completion of a purpose-designed adherence to face and body photoprotection measure, where a total score was calculated by considering the relative protection afforded by different protection combinations, plus a single-item to measure avoidance of going outside as a means of protection (see Canfield et al., 2019). Participants completed a series of validated self-report questionnaires to measure self-efficacy, automaticity, intention to protect, illness perceptions (consequences, timeline, personal control of XP, photoprotection control of XP, treatment control, identity, negative emotional representation, and perceived understanding), perceived effectiveness and necessity of protection, concerns about protection, and social support. Most measured variables (self-efficacy, automaticity, intention, effectiveness, necessity, concerns, and illness perceptions: consequences, personal and photoprotection control) were associated with a greater likelihood of using better face and body protection, in the expected directions (between-participant analysis using ordinal logistic regression). Confirming the existence of a protection-wellbeing trade-off, XP-related distress (negative emotional consequences) was related to greater avoidance of going outside but not protection when outside.

**Table 1.** Data sources and how they were used for personalisation and tailoring in XPAND.

Data source	When data was obtained from participant	Sub-categories derived from data source	How data source was used	Decision point: a-priori (before delivery), in-session (iterative)
UVR daily diary (7 weeks)	Phase I	Average daily time spent outdoors Photoprotective clothing combinations used when outdoors, and proportion of outdoor time protected by each Frequency of sunscreen application and proportion of outdoor time protected by sunscreen	To generate individual risk and behaviour profile, per participant (a-priori), which informed feedback/discussion of behaviour and risk in session 1 and led to generation of options for behavioural improvement (collaboration between facilitator and participant) Application of standardised content (SMART goal setting: behaviour), including behaviours/combinations already being used some of the time vs. the need for new behaviours/combinations to be added to behavioural repertoire to achieve higher-level protection; selection of behavioural goal each session	A-priori (tentative) and then confirmed with participant following presentation of feedback in session 1 Updated during goal setting/ review activity during each subsequent session
n-of-1 study (7 weeks)	Phase I	Dynamic logistic regression: intra-individual correlates of Daily Photoprotection Scale (newly developed scale, derived from UVR daily diary data) Spearman’s correlations between self-reported protection (0-100) and EMA variables (e.g. effort, mood) Absolute levels of EMA variables Environmental/cue-based (time of day, weekend vs. weekday, weather/sunny, physical symptoms, perceived need for protection) Self-regulatory (effort, barriers, planning, self-efficacy) Motivational (importance, motivation, confidence) Emotional (mood, stress, negative thoughts, mental exhaustion, active/energy, quality of life) Social (social support, missing out, self-consciousness)	To inform/prompt discussion in session 1 around relevant barriers to achieving better/more consistent protection across situations/contexts, including providing feedback on observed patterns and seeking confirmation/disconfirmation and/or further detail on how each barrier is linked to protection (i.e. nature of barrier and how/why it manifests) – this conversation is started in session 1 and elaborated in later sessions, as each barrier is addressed To select which personalised modules would be delivered To inform the likely strategies needed within chosen modules, given observed direction of relationship (e.g. stress as cause or consequence of better/worse protection)	A-priori (tentative) and then confirmed with participant in session 1 Referred back to each session if/when new barriers are identified – compare to patterns identified in Phase I
Qualitative interviews	Phase I	Identification of three types of responses to photoprotection, based on description of protection used and balance between medical and psychosocial priorities: ‘dominated’, ‘integrated’, and ‘resistant’ Personal attributions for level of protection employed Identified barriers to using better protection	To inform/prompt discussion in session 1 around relevant barriers to achieving better/more consistent protection across situations/contexts To select which personalised modules would be delivered To inform the order in which personalised modules would be delivered (based on apparent priority/significance of identified barriers) To inform the specific examples that strategies within each module were applied to (elaborated in session)	A-priori (tentative) and then confirmed with participant in session 1 Referred back to each session if/when new barriers are identified – link to attributions/ barriers identified in Phase I
Cross-sectional survey	Phase I	Adherence to face and body protection behaviours (newly developed scale) Raw/subscale scores on correlates of behaviour Necessity and concerns Illness perceptions and emotional representation Intention Self-efficacy Automaticity Social support Psychological wellbeing	To inform/prompt discussion in session 1 around relevant barriers to achieving better/more consistent protection across situations/contexts Application of standardised content (SMART goal setting: behaviour), including behaviours/ combinations already being used some of the time vs. the need for new behaviours/combinations to be added to behavioural repertoire to achieve higher-level protection; selection of behavioural goal each session To select which personalised modules would be delivered	A-priori (tentative) and then confirmed with participant in session 1
Profiling questionnaire	Trial baseline	Behaviour (frequency of protection, frequency of sunscreen application, time spent outdoors) Individual attributions for protection/impact on protection Necessity (in different weather conditions) Personal and treatment control Personal susceptibility and fatalistic beliefs (e.g. cancer will get me one day regardless of behaviour) Emotional impact, disruption, worry about others’ reactions Automaticity, use of visual cues/weather to protect Self-efficacy Importance and motivation Support and openness with others (disclosure) Planning (action/coping)	To inform/prompt discussion in session 1 around relevant barriers to achieving better/more consistent protection across situations/contexts Application of standardised content (SMART goal setting: behaviour), including behaviours/ combinations already being used some of the time vs. the need for new behaviours/combinations to be added to behavioural repertoire to achieve higher-level protection; selection of behavioural goal each session To select which personalised modules would be delivered (drivers of behaviour)	A-priori (tentative) and then confirmed with participant in session 1
Response to intervention	In session	Response to feedback (behaviour and/or barriers to protection, as discussed in session 1) Response/reaction to content (e.g. need for further detail on a given topic, return to same topic after change has been attempted, coverage of one topic naturally leads into discussion of another topic) Self-reported importance, confidence, willingness for behaviour change, as assessed when completing SMART goal setting/action planning section of each session New/unanticipated barriers emerge as behaviour change is attempted or achieved	To select which personalised modules would be delivered To inform the order in which personalised modules would be delivered (based on which barriers are perceived as posing more threat to behaviour change, and personal preferences/willingness to discuss) To adapt standardised content to the specific nature of experienced and/or anticipated barriers	In session/ iterative

**Table 2.** Use of data in each module.

Module	Personalisation (allocation)		Tailoring (adaptation)
	Data source	Relevant variables/indicators leading to decision point	
Stress and Mood	N-of-1	Stress Mental exhaustion Mood Negative thoughts Active (energy) Quality of life	Specification of direction and nature of relationship between stress/mood and protection, including reference to other variables (e.g. availability of psychological resources, positive affect resulting from protecting oneself well) Application of stress/mood management strategies adapted according to individual triggers/ source (e.g. excess of depleting activities vs. absence of nourishing activities); degree of control and suitability to problem solving/ planning vs. need for self-care; cognitive vs. behavioural symptoms and preference for cognitive vs. behavioural strategies; management of acute/reactive symptoms vs. reducing vulnerability Goal options: How will I boost my resources to minimise stress/ reactivity? Stress/mood management to minimise impact of stress/low mood on photoprotection (thinking and/or behaviour) Action planning: ‘To ensure that I’m at my best to achieve my goal, I will boost my resources by … ’ Coping planning: ‘If I am feeling stressed, then I will … ’; ‘If I am feeling down or notice an unpleasant thought, then I will … [behavioural strategy]/remind myself that … [thinking strategy]’
	Qualitative interviews	Negative emotional responses to XP and photoprotection demands Negative emotional consequences of high adherence were a key feature of the ‘dominated’ group	
	Cross-sectional survey	Emotional representation Psychological wellbeing	
	Baseline profiling questionnaire	Emotional effects of XP and photoprotection	
	In-session/dynamic	Stress or negative mood (or other relevant emotional states) emerge … in session 1 discussion as reason for differing protection across situations/contexts as anticipated barriers when completing coping planning as reasons for low self-reported confidence, willingness, or importance during goal setting/action planning during goal review as experienced barriers to achieving photoprotection goal as consequences of behaviour change attempt/achievement	
Acceptance and Willingness	N-of-1	Importance Motivation Missing out	Identification of personal values and generation of personal past examples of when values have been used to prompt behaviour, even when motivation is reduced, or the immediate effects are undesirable Consideration of how to align personal values with protection (vs. protection as a barrier to engagement with values), including reasons for protection that are broader than the ‘facts’ related to cancer risk and skin damage (personal ‘carrots’ to supplement known ‘sticks’) Application of values-based and acceptance/willingness strategies, adapted to personal situation Goal options: Same goal framed explicitly in relation to values: does this make a difference? New goal to bring behaviour/protection into line with long-term values. Action planning: value-based rewards, meaning/value-based reminders, ‘The values underlying my goal are … ’; ‘To meet my goal, I will need to accept that … ’ Coping planning: ‘If my ‘in the moment’ motivation is flagging, then I will remind myself that … ’; ‘I am willing to … even if … I will do this by … ’
	Qualitative interviews	‘Resistant’ adherence group described many of the most powerful barriers to photoprotection (e.g. prioritisation of other things in life, low social support, stigma, concealment of XP)	
	Cross-sectional survey	Importance Intention to photoprotect Intention to avoid going outdoors	
	Baseline profiling questionnaire	Importance of protection compared to other life priorities Disruption to everyday life Worry about other people’s reactions	
	In-session/dynamic	Resistance to behaviour change (initial and/or to go further once some improvement has been made) emerges as a barrier to ongoing change (anticipated: coping planning; experienced: goal setting/goal review) Low self-reported willingness for behaviour change (as per current goal), as assessed during the goal setting/action planning section of each session Perceived negative consequences of improved photoprotection (e.g. missing out, low mood) result in reduced motivation/ willingness for further change	
Social support and disclosure	N-of-1	Level of support	Discussion of relevant magazine content and how it matches/ applies to their individual situation; identification of personal sources of practical and emotional support and perceived helpfulness/desire for change Application of effective communication strategies, adapted according to type of support needed (practical vs. emotional, and specific needs within each), who they are seeking support from, level of disclosure needed/comfortable with (which may differ across situations and support people), past experiences with disclosure, perceived pros and cons of disclosure Depending on their current level of openness, the full disclosure sub-module may be given, or disclosure mentioned only minimally in the context of support seeking Goal options: Mobilise my friends and family to support my photoprotection goal Action planning: Who will help me? What will they do? Option of setting a separate SMART goal and action plan for social support/ disclosure Coping planning: ‘If I feel I need more practical support to achieve my protection goal, then I will … ’; ‘If I feel that there is not enough time to talk about XP to my friends, then I will … ’
	Qualitative interviews	Experience of support was influenced by interactional processes; well-intentioned support can be perceived as unhelpful ‘Toxic support’ where the taking of risks with UVR exposure was encouraged Importance of helpful practical social support in facilitating photoprotection	
	Cross-sectional survey	Level of support Satisfaction with support	
	Baseline profiling questionnaire	Level of support Perceived usefulness of support Openness about XP/ photoprotection (e.g. with friends, colleagues)	
	In-session/dynamic	Absence of social support (or limited/ unhelpful support) or reluctance to disclose emerges … in session 1 discussion as reason for differing protection across situations/contexts as anticipated barriers when completing coping planning as reason for low self-reported confidence, willingness, or importance during goal setting/action planning during goal review as an experienced barrier to achieving photoprotection goal Reluctance to specify ‘who could help me’ in the SMART goal setting/action planning section of each session (although this doesn’t necessarily indicate need for improved support – some goals do not require the involvement of others) Difficulties with relationships emerge as a consequence of behaviour change attempt/ achievement and/or disclosure/request for support	
Appearance concerns	N-of-1	Self-consciousness	Discussion of relevant magazine content and how it matches/ applies to their individual situation; identification of personal concerns (e.g. may be related to appearance as a result of condition and/or appearance when using good protection) and prior experiences of reactions from others (negative or positive) Application of management strategies, adapted according to nature of concerns, preference for cognitive or behavioural strategies, willingness to experience feelings of self-consciousness (this may shift over intervention), existing skill and comfort in social situations, past experiences Goal options: Strategies to minimise impact of worries about looking different on photoprotection (thinking and/or behaviour) Coping planning: ‘If I don’t want to protect as it makes me look different, then I will remind myself that … ’; ‘If I’m feeling self -conscious about my protection, then I will distract myself by … ’
	Qualitative interviews	Experiences of stigma and appearance concerns (e.g. freckling and looking different whilst protecting)	
	Cross-sectional survey	Worry about other people’s reactions	
	Baseline profiling questionnaire	Worry about other people’s reactions	
	In-session/dynamic	Appearance concerns emerge … in session 1 discussion as reason for differing protection across situations/contexts as anticipated barrier when completing coping planning as reason for low self-reported confidence, willingness, or importance during goal setting/action planning during goal review as experienced barrier to achieving photoprotection goal as consequences of behaviour change attempt/achievement	
Necessity Sub-modules: Underestimating environmental risk Underestimating risk in the absence of burn or skin changes Use of symptoms to guide protection Underestimating cancer risk related to having XP Fatalistic belief that cancer is inevitable regardless of behaviour Doubts about effectiveness of protection Extreme confidence in clinical treatment	N-of-1	Weather/sunny Perceived need for protection (risk perception)	Within each personalised necessity sub-module (allocated by matching to identified need from profiling questionnaire): Elicitation (brief) of nature/details of necessity-related doubts, focusing on personal views rather than textbook facts or expected medical response, selectively reinforcing ‘necessity talk’ and exceptions, linking to personal reasons for protection even in the presence of doubts (‘carrots’) – motivational interviewing Discussion of relevant magazine content and how it matches their personal understanding/behaviour Application of habit formation (cues to action) and willingness strategies, adapted according to the specifics of the target necessity belief, focusing on the replacement and/or supplementation of contingent (e.g. weather/season) cues for protection and practicing willingness (linked to personal reasons/values) in the absence of strong motivation/belief in the necessity of protecting and/or persistence of uncertainty Goal options: Thinking strategies to minimise the impact of doubts about why protection is important/necessary Coping planning: ‘If it is cloudy and I feel less like wearing my sunscreen, then I will remind myself that … ’; ‘If I’m wondering whether protection is really worthwhile, then I will remind myself that … ’;
	Qualitative interviews	Strong impact of seasonal and weather changes on perceptions of risk and photoprotection levels Believing cancer to be inevitable (leading to poorer protection) Doubts about the effectiveness of sunscreen Use of symptoms to guide photoprotection	
	Cross-sectional survey	BIPQ: consequences BIPQ: duration BIPQ: personal control of XP BIPQ: photoprotection control of XP BIPQ: treatment control BIPQ: illness concern Beliefs about photoprotection: necessity Beliefs about photoprotection: concerns	
	Baseline profiling questionnaire	BIPQ: photoprotection control of XP BIPQ: treatment control BIPQ: personal control of XP Necessity to protect when cloudy Necessity to protect in winter Necessity to protect if outdoors for short time Level of skin cancer risk Fatalistic beliefs Use of weather to make judgements	
	In-session/dynamic	Doubts about effectiveness/necessity of protection emerge … in session 1 discussion as reason for differing protection across situations/ contexts as anticipated barriers when completing coping planning as reason for low self-reported confidence, willingness, or importance during goal setting/action planning during goal review as experienced barriers to achieving photoprotection goal if/when unfavourable clinical results are received (e.g. skin cancer diagnosis despite improvements in protection; either during course of intervention or anticipated as future barrier to maintenance)	
Self-regulation **(core content –** delivered to all)	N-of-1	Effort Barriers Planning Confidence	SMART goal setting, action planning, coping planning all adapted according to current behaviour and identified options for improvement, nature of other changes/support needed to achieve change, and nature of anticipated and/or experienced barriers (informed by personalised module content) Self-monitoring to track progress, with method (paper-based record, phone app) and frequency (e.g. daily vs. weekly check-in) tailored to individual preference Problem solving mini-module delivered when appropriate and tailored to a specific situation identified by participant (this could occur in the context of any of the personalised modules)
	Qualitative interviews	Provided detail on the range of barriers experienced	
	Cross-sectional survey	Self-efficacy for photoprotection Self-efficacy for avoiding going outside	
	Baseline profiling questionnaire	Action planning Coping planning	
	In-session/dynamic	Use/usefulness of goal setting, action planning, coping planning, self-monitoring, as determined through goal review at beginning of each session Identification of lack of planning/ preparation as a reason for non-achievement or partial achievement of behavioural goal	
Habit formation (**core content** – delivered to all)	N-of-1	Weather/sunny (cue to action) Physical symptoms (cue to action)	Discussion of existing individual routines and schedules within which protection was needed, and application of habit formation strategies (e.g. cues to action) adapted to the specific characteristics of their routines (including changing routines such as alternating shifts). In the absence of existing routines or stable cues for protection, personally-relevant options were considered Text messages: selection of spacing and timing of messages to best match schedule; matching of content to most recently-delivered personalised module or barrier targeted in coping planning; use of individual examples in text messages
	Qualitative interviews	Experiences of the ‘integrated’ group highlighted the importance of habit and routines	
	Cross-sectional survey	Automaticity of photoprotection Automaticity of avoiding going outside	
	Baseline profiling questionnaire	Automaticity of photoprotection Purposeful placement of protective items as reminders (cues to action)	
	In-session/dynamic	Use of cues to action (vs. forgetting in the absence of cues to action) Photoprotection routine as facilitator/absence of routine as barrier to improved protection (anticipated: coping planning; experienced: goal review)	

Notes: XP: xeroderma pigmentosum; BIPQ: brief illness perception questionnaire; self-regulation and habit formation content was delivered to all participants, so no personalisation occurred; however, tailoring of the standardised content was undertaken. In this case, the points listed under ‘data source’ and ‘relevant variables/indicators leading to decision point’ refer to when and the intensity/frequency/emphasis given to these strategies rather than whether or not they were given.

The reasons for drawing on multiple data sources included that not all intervention participants had previously participated in the phase I studies (i.e. some had been diagnosed since 2016 and their suitability for intervention was identified by other means), and for those who did, some did not participate in all sub-studies (e.g. some completed the qualitative interview but not n-of-1 study). Further, within the n-of-1 study, there were also differences in the level of information available for each participant, depending on the number of outdoor occasions recorded over the 7-week data collection period, and the level of variation in protection behaviour, self-reported adherence (0–100), and ecological momentary assessment (EMA) predictors, the lack of which precluded dynamic modelling and/or conduct of Spearman’s correlations in some cases (Sainsbury et al., 2018b). To ensure that all available information was used, the absolute levels of EMA predictors in the n-of-1 study were, therefore, also considered, as, even in the absence of variation with protection, the measured constructs could still represent barriers to motivation and/or protection, either currently or in the future, if not addressed.

Additionally, a baseline profiling questionnaire (not used in phase I) was designed for the trial. Although this questionnaire did not provide information on the covariation of predictors with protection behaviour, it was available for all intervention participants and was the assessment conducted most proximally to intervention delivery. It could, therefore, be used to identify probable changes in protection and barriers since phase I, or fill gaps for participants who did not have complete phase I data. In addition, it provided detailed information on specific necessity beliefs (e.g. doubts about the effectiveness of sunscreen/clothing protection; doubts about the UVR exposure-cancer link; necessity of protection in different weather conditions), which could be mapped to content within the necessity module (7 sub-modules targeting different beliefs/doubts), as this level of detail was not available from phase I. This latter approach is consistent with several previous successful interventions that have drawn on belief-based data from the illness perceptions questionnaire to individualise behaviour change content (e.g. Petrie, Cameron, Ellis, Buick, & Weinman, 2002, 2012).

### Decision points

A ‘decision point’ is any point prior to, or throughout, the intervention when a decision about content delivery for the individual is made, prompted by identification of a relevant trigger for treatment change (which could include pre-specified ‘if-then’ rules, and/or iteratively-identified indications). The XPAND intervention was structured such that sessions 1, 6, and 7 predominantly contained core content and strategies delivered to all participants (with tailoring of that content), while sessions 2–5 contained both core content (e.g. goal setting and review, action and coping planning, habit formation) and personalised content (i.e. allocated based on assessment of need/relevance, and then tailored within each allocated module). Both personalisation and tailoring processes involved dynamic (vs. static/one-off) decisions, whereby a-priori recommendations for behavioural goals and relevant modules derived from the various data sources were confirmed or disconfirmed in collaboration with the participant and reviewed and refined iteratively as various topics were covered, and/or new barriers emerged as behaviour change was attempted or achieved. This could occur at any point throughout intervention delivery, although specific decision points included the following:

- Session 1 discussion: identification of barriers/reasons for less than ideal or inconsistent protection, following individual feedback on risk and phase I behaviour (e.g. percentage of outdoor time protected using each DPS category; percentage of outdoor time protected by sunscreen)
- Goal review: experienced barriers to improved protection/goal achievement since the last session
- Coping planning: anticipated barriers before the next session
- SMART goal setting: low self-reports of importance, confidence, or willingness for behaviour change (assessed during goal setting section of each session), which prompted discussion to identify reasons and the potential need for changes to the goal and/or further coping planning to overcome barriers

### Personalisation (allocation of modules to individuals)

Prior to session 1, all available data for each participant (phase I + baseline profiling questionnaire) was triangulated to make a-priori recommendations on the allocation of modules. This involved the creation of two profiles per person: the first contained behavioural information, which was linked to personalised risk associated with less than ideal protection; and the second contained a summary of the environmental, belief-based, self-regulatory, and emotional data that indicated the likely barriers to protection (referred to as the ‘psychological profile’). The n-of-1 results and qualitative interview (where available) were used to suggest relevant modules to target the identified barriers (see Table 2); qualitative data were also used to provide additional insights/details on why particular patterns may exist. The cross-sectional survey data and baseline profiling questionnaire were then consulted to determine whether any additional variables may be relevant or whether behaviour or levels of constructs that were measured in both (e.g. self-efficacy, automaticity) had changed since phase I. The profiles were compiled by two researchers (KS, JWa) who were involved in phase I and the design and delivery of XPAND. Key considerations during this collaborative process included the degree of consistency between the phase I data and baseline profiling questionnaire (not all constructs were measured at both times), whether the various data sources pointed to similar barriers, and whether the quantitative data was in agreement with the patient’s experience and attributions from the qualitative interview. All trial participants consented to use of their prior (phase I) data when providing informed consent for the trial.

Session 1 was a key decision point for subsequent personalisation (and tailoring), on the levels of behaviour and multiple theoretical constructs. Individual behavioural/risk feedback was presented to the participant and used to prompt a discussion about behavioural options for achieving improvements in protection, reasons for using their chosen protection, and why protection varied across situations and contexts (as a way of accessing barriers). The former could include the addition of new behaviours not already being used and/or using existing behaviours in novel combinations and/or more consistently across situations, from which a behavioural SMART goal was later generated. Although personalisation/tailoring on the basis of behaviour is not uncommon, the complexity of photoprotection behaviour in XP and extent of available assessment data meant that the individual feedback linked to the provision of normative (i.e. most people had room for improvement, at least some of the time) and risk-based information, and various options for improvement, per person, was far more detailed and led to more numerous decision points for personalisation than in previous tailored interventions.

The participant’s identification of barriers to protection through the session 1 discussion was infused by the intervention facilitator with details from the psychological profile – this could take the form of the participant identifying barriers/reasons that were consistent with the phase I data/ profiling questionnaire, which were then reinforced with reference to phase I data (both within- and between-participant findings), or discussion was prompted/supplemented by potential barriers not mentioned by the participant. In the latter case, the participant was asked to consider whether the identified patterns matched their personal experience and made sense in the context of their understanding of their protection, and whether they could give further details on the nature/reasons for the identified patterns. If a participant was struggling to generate attributions for their varied protection, the facilitator could also prompt discussion by referring to between-participant findings (e.g. ‘In phase I, some people said … ’ or ‘In phase I, there was a strong relationship between lesser protection and … ’). The advantage of using both quantitative (n-of-1) and qualitative (interview) data to inform this conversation was that consciously-known attributions could be supplemented with new insights, derived from the participant offering a personal interpretation (that they may not have previously been aware of) of the observed data patterns. The facilitator indicated to the participant that XPAND was designed to target a range of different barriers identified in phase I, that everybody’s experience was different, and that together they would decide the relevant focus. Session 1 was largely manualised, although the collaborative conduct of the session drew heavily on aspects of personalised care planning and shared decision-making interventions, resulting in a rich evidence-base which set the scene for the personalisation of subsequent sessions.

A decision about which personalised module would be delivered in session 2 was made collaboratively at the conclusion of the session, following the setting of a behavioural SMART goal and completion of the action and coping planning activity. The facilitator made a suggestion based on the a-priori profiles and session 1 discussion, to which the participant could agree or suggest a different starting point. When doubts about necessity or reduced acceptance, motivation, or willingness to protect were identified as barriers, these were prioritised for session 2, as they were considered central barriers that would likely require greater treatment than was included in session 1, with shifts needed before a participant may be open to receiving volitional strategies. Decisions about subsequent modules were made in a similar manner, referring back to barriers identified in session 1, and any newly-identified barriers as behaviour change was attempted or achieved. More detailed discussions in later modules around the direction of identified relationships with protection (e.g. whether increased stress prompts better/worse protection or whether increased/decreased stress is the consequence of better/worse protection) and the influence of other factors on relationships could also suggest additional possibilities. Where several possibilities were available, weekly peer supervision between the three intervention facilitators (KS, JWa, LF) was used to discuss options and make recommendations for content and order, and the original tentative agendas for all participants were iteratively-refined as the intervention progressed.

### Tailoring (adaptation of content)

The goal-oriented nature of XPAND meant that participants reviewed and set goals each session, so that improved protection was cumulative across the 7 sessions. Consequently, one level of tailoring involved the adaptation of content (core and personalised) to match the current behavioural goal (e.g. discussion of how appearance concerns get in the way of wearing a face buff, which may be different from other forms of protection). After session 1, participants were given the option of selecting a new goal (if they had achieved the previous one) or keeping the same goal while adding action plans specific to the personalised module to test whether this made it easier to achieve in the coming week. For example, in the cases of acceptance and stress, respectively, they could keep the same goal but see whether framing it explicitly in terms of their values/non-health-related reasons for protection, or while using strategies to minimise stress/boost resources, made a difference to enactment.

Similarly, coping plans were generated for each type of barrier (i.e. personalised module) using a pre-specified format. For example, for necessity beliefs and social support, respectively, coping plans were linked to specific barriers (‘if’ statements) such as, ‘If it is cloudy and I am doubting the need to protect myself [insert specific form of protection]’ or ‘If I feel I need more practical support to achieve my protection goal’. Depending on the nature of the strategies covered in each personalised module, relevant ‘then’ statements were provided as the stem for coping plans (e.g. ‘then I will remind myself that … ’ or ‘then I will distract myself by … ’). Participants could, of course, add their own unique barriers/solutions to this list. A volitional help sheet containing ‘if’ and ‘then’ statements derived from the phase I data was available for reference, if participants were struggling to anticipate likely barriers or generate their own solutions.

As behaviour change was achieved, the facilitator could also prompt a return to the personalised behaviour and risk feedback given in session 1, as it was possible that, with reductions in barriers and/or increases in motivation or self-efficacy resulting from goal achievement, participants would be willing to consider adding forms of protection that were not considered an option at the start. This could mean that new barriers emerged or that previously-delivered topics required reiteration with specific reference to the new behavioural goal. Another core strategy, problem-solving, formed a mini-module, which was applied as needed during sessions 2–5 and tailored to a specific situation identified by each participant.

Similarly, strategies for habit formation were adapted to the individual; for example, by discussing the specifics of existing routines (e.g. work, home, travel) and selecting appropriate cues to action for each relevant context in which photoprotection was required. Within the text messages sent to participants between sessions, tailoring strategies included mapping content to the most recently-delivered personalised module and drawing on personal examples to further increase the relevance of received texts, and allowing the participant to choose the spacing and timing of messages to prompt their behaviour at the most relevant times in their individual schedules.

Within each personalised module, tailoring also took the form of matching the suggested cognitive and behavioural strategies to reduce/manage the relevant barrier to participant-generated examples and their specific experiences and beliefs (e.g. the nature of the doubt, trigger for stress; see Table 2). These were balanced with prompts to consider drawing on motivation (using the ACT-informed ‘carrots and sticks’ metaphor introduced in session 1; where ‘carrots’ represent future-focused, values- and benefit-based reasons for protection, and ‘sticks’ represent avoidance-based motivations, such as avoidance of skin cancer, and/or the strategy of motivating oneself to change by using self-criticism) and willingness to engage in protective behaviour, even in the presence of barriers. Depending on the preference and responsiveness of each participant to these different ways of achieving behaviour change (reducing barriers vs. boosting motivation), the facilitator could also tailor content and discussion to differentially emphasise one or the other at different points.

Finally, after delivering the majority of the active behaviour change content in sessions 1-5, sessions 6 and 7 were focused on the *maintenance* of behaviour change. Participants were prompted to reflect on each identified/addressed barrier and the changes they had made, long-term or anticipated future barriers to continued motivation and/or behaviour, and how they would maintain their improved protection if/when something happened to threaten their progress. Reflection on personal behavioural and other changes was an important component of these sessions (akin to repeat assessment and used to dynamically tailor information) as, conceptually, the relevance of the maintenance strategies would be heavily dependent on the match with current behaviour and thinking, which was expected to be different from that assessed and discussed in session 1 (i.e. if the intervention had worked to achieve intra-individual improvements). Previously-delivered content/strategies were then reiterated and successful enactment despite barriers was reinforced, again tailored specifically to the unique context, experiences, routines, strengths, past successes (as discussed/observed throughout intervention delivery), and current/new state of behaviour for each person. The addition of maintenance-relevant strategies to the accumulation of action and coping plans from earlier sessions formed an individually-tailored volitional help sheet, which could be referred to, as needed, in the future (e.g. as a periodic reminder/refresher or relapse prevention during personally-risky situations).

## Discussion

This paper describes the personalisation and tailoring of ‘XPAND’, a purpose-designed, evidence- and theory-based behaviour change intervention to improve adherence to photoprotection recommendations in patients with the rare disease, XP. Drawing on evidence from previously-successful tailored interventions, XPAND was designed to utilise dynamic tailoring based on behaviour and multiple theoretical variables, applied to the allocation of intervention modules (personalisation) and adaptation of module content and text messages (tailoring) to the individual participants. This involved both a-priori and iterative decision-making, based on data from a series of phase I studies in the target population and individual response to the intervention. The personalisation and tailoring process was complemented by core self-regulatory and habit formation content, delivered to all participants.

Key factors in the choice of whether, and to what extent, tailoring and personalisation are feasible are typically related to the nature of the condition, behaviour (complexity), and population (size), and trade-offs between reach and efficacy, and effort and cost. While the process described here may not be feasible for more common conditions and behaviours, in the context of a very rare disease, where the individual and financial burden of illness and treatment is high, and the usual method of piloting and refining an intervention was limited, the current approach was deemed both feasible and necessary to achieve the project aims. One particular tension arose from the need to do everything possible to ensure effectiveness (e.g. intensity, resources, choice of techniques and facilitators, level of tailoring/personalisation), as we only had one opportunity to ‘get it right’, while also holding the goal of eventually integrating XPAND into routine clinical practice.

Consequently, the choice of intervention facilitators in the trial was an important consideration in intervention planning. Reference to the Health Behaviour Change Competency Framework (Dixon & Johnston, 2010) suggests that XPAND is predominantly a medium intensity intervention, defined by the existence of a manual to guide intervention delivery but which offers the practitioner some flexibility in delivery. Although some competencies for a high intensity intervention were also needed^1^, personalisation (i.e. allocation of modules) and tailoring decisions (i.e. adaptation) were often not implemented spontaneously but were made either prior to commencement of the intervention or in consultation between facilitators in weekly supervision meetings. A multidisciplinary approach, including a clinical psychologist (KS), a health psychologist (JWa), and a rare disease nurse (LF; who previously specialised in another rare skin disease) was decided on, as it provided an acceptable means to bridging the gap between the differing professional backgrounds and competencies of the research and clinical teams. In particular, the inclusion of a nurse in the intervention design and delivery process meant that any difficulties arising from their lower level psychological knowledge (compared to the psychologists) could be addressed; for example, via the provision of relevant reading/training to upskill, such as was done with motivational interviewing, and/or limiting the complexity of the strategies or explanation included in the intervention, so they remained within the skill set. Equally important, this approach also ensured that the patient-centred intention of the intervention (e.g. that both intervention delivery and the suggested strategies were seen as relevant and fit within their lives, and could be adapted to preferences, needs, knowledge, and ability, rather than requiring a high level of existing psychological knowledge) was maintained.

Assuming that XPAND is effective (trial results to be published elsewhere), the clinical roll-out will be led by the XP Clinical Nurse Specialists (CNSs). Knowing this from the outset, their involvement, plus that of the broader clinical and PPI teams, throughout all stages of the XP project, was another essential part of ensuring that the developed intervention could be feasibly integrated into the existing care pathway. While the CNSs have not had extensive behaviour change training, the nature of their roles within the NHS already involve ‘detailed, holistic needs assessment; individualised care planning; provision of individualised information and advice to patients and their families/carers including, where needed, direct phone contact outside of scheduled appointments; minimisation of both the clinical and the psychosocial effects of the patient’s condition and/or its treatment’ (Vidall, Barlow, Crowe, Harrison, & Young, 2011, p. 3). Thus, it was felt that the structure of the highly specialised XP service, and the CNSs vast knowledge of XP and their relationship with patients and existing skills in communicating 1:1 with patients to deliver individualised treatment protocols offered good mechanisms for tailoring that could be supplemented by use of the XPAND manual and specific behaviour change, motivational interviewing, and ACT-based training (for technical and stylistic purposes, respectively).

A strength of the current approach was the comprehensiveness of the formative data available, from which the a-priori behavioural and psychological profiles were generated and used to inform tailoring and personalisation decisions. Although highly time-consuming and resource-intensive, this step was initially essential to inform the development of the intervention content and methods, in the absence of any prior psychosocial research in XP and the lack of effective sun protection interventions in the general population or other high-risk groups on which to base intervention decisions. Only following the understanding that came from this formative research and consequent capacity to develop a relevant and needs-responsive intervention was the data used for personalisation and tailoring. Thus, the time taken for the whole process would mostly have been necessary, whether personalisation and tailoring were used or not. Considering the potential translation of the current work to other rare conditions, many of which are also characterised by a lack of psychosocial and intervention research, it is likely that significant investment (time and financial) will be needed for intervention development. Done with the ongoing contribution of stakeholders and within the framework that the NHS provides (i.e. highly specialised services and use of CNSs to coordinate care; and for which there are likely equivalent services in other countries), we believe that the time and resources required for this approach (development and personalisation/tailoring) are not unreasonable, if the need for research and the addition of evidence-based behaviour change support is strong. It is also likely that front-heavy resource allocation will have later pay-offs associated with increased efficiency and effectiveness of the care delivered.

Having said this, we concede that this ‘gold standard’ approach will not always be possible. Instead, we would suggest that much like experienced clinicians often match and creatively adapt transdiagnostic approaches that are at least partially manualised (e.g. ACT and CBT from the clinical psychology field, or self-regulation and habit formation strategies from the health psychology field) to individual patient presentations, it may be possible to adapt parts of XPAND (content and methods) to other populations requiring sun protection or other rare diseases where less information about individual barriers is available. This could be achieved, for example, by mapping the disease-specific knowledge and insights about illness adjustment and reasons why patients do or do not adhere to treatment recommendations already held by CNSs or other clinical experts to a list of potential intervention strategies that have been selected to target common barriers. This could be supplemented by less time-intensive data collection methods in the target population (e.g. questionnaires and short answer elicitation questions), as well as the early use of self-monitoring techniques within the intervention to gain an understanding of behavioural variability and patient attributions for such variability (note: self-monitoring is an effective behaviour change method, over and above its use for data collection). Following intervention design/adaptation, the use of a purpose-designed screening tool to determine the relevance of the selected strategies to individuals within the target population could then provide a short-cut to the allocation of content. While not as comprehensive as what we have presented here, it is likely that some level of patient-focused evidence-gathering and subsequent personalisation and tailoring is better than a one-size fits all approach.

Indeed, moving forward, it is unlikely that the XP clinical team will have access to the same level of in-depth, standardised information for new patients entering the service. A question for a later stage of implementation research that is also relevant here is, therefore, whether a lower level of information can be used to achieve similar differentiation and change. This was one reason for the development of the baseline profiling questionnaire, as it provides a simpler and more-proximal assessment of potential drivers of photoprotection behaviour, which will be used as a screening tool by the clinical team, and supplemented by the CNSs knowledge of patients and clinical information gained via the regular assessment procedures already used in the XP clinic. The provision of a manualised programme of core behaviour change components (e.g. self-regulation, habit, and willingness content, with inclusion of a magazine and worksheets) that are likely to be relevant to most XP patients, with options for tailoring/personalisation if specific barriers emerge, as well as ongoing contact between the research and clinical teams beyond the trial, and planned training workshops and demonstration videos will, together, hopefully ensure that effectiveness and acceptability are maintained in a real-life setting.

XPAND represents a unique example of the development and delivery of a personalised and tailored behaviour change intervention; we are unaware of any prior attempt on the scale of which we have achieved. Dynamic, multi-level personalisation and tailoring based on mixed-methods allowed for insights and decision-making not possible with cross-sectional quantitative or qualitative methods alone. While our aim was not to compare to non-tailored delivery, or suggest that this method could be used in all fields, the potential for extensive tailoring here closely mimics the delivery of clinical care in the healthcare setting – one of the conditions under which personalised care planning was most effective (Coulter et al., 2015). The difference is that, by conducting the XP project, future CNS-delivered care can be infused with evidence-based behaviour change components to target individual barriers, and with clear processes to identify need and monitor change/progress. Where rare diseases are often neglected in behaviour change and psychological treatment research, we hope that the data collection and personalisation/tailoring methods described here and throughout our phase I studies, as well as recommendations for lower-level adaptation, may offer solutions that will aid the future development of increased support options for people with rare diseases.

## Data Availability

Ethical approval for the trial of XPAND has been received from West London & GTAC REC 17/LO/2110.

## Note

1 For example, knowledge of behaviour change models and the ability to apply them flexibly with a patient, while remaining adherent to the chosen model; capacity to adapt interventions to client need (including personal style and social and cultural difference), and to select and apply the most appropriate intervention method; and the use of specific BCTs for motivation, action, and the prompted/cued routes to behaviour.

## Data availability statement

Please contact authors to request access to any of the data referred to in this paper

## References

[CIT0001] Anderson, R., Walburn, J., & Morgan, M. (2017). Experiences of stigma over the lifetime of people with xeroderma pigmentosum: A qualitative interview study in the United Kingdom. *Journal of Health Psychology*, *24*, 2031–2041. doi:10.1177/1359105317714643 28810476

[CIT0002] Anderson, R., Walburn, J., & Morgan, M. (2019). Approaches to photoprotection and normalization in highly adherent families of children with xeroderma pigmentosum in the United Kingdom. *Qualitative Health Research*, *30*(8), 1275–1286. doi:10.1177/1049732319826561 30741094

[CIT0003] Bradford, P. T., Goldstein, A. M., Tamura, D., Khan, S. G., Ueda, T., Boyle, J., … Kraemer, K. H. (2011). Cancer and neurologic degeneration in xeroderma pigmentosum: Long term follow-up characterises the role of DNA repair. *Journal of Medical Genetics*, *48*(3), 168–176. doi:10.1136/jmg.2010.083022 21097776 PMC3235003

[CIT0004] Canfield, M., Norton, S., Walburn, J., Morrison-Bowen, N., Sainsbury, K., Araujo-Soares, V., … Weinman, J. (2019). Facial photoprotection in xeroderma pigmentosum (XP) patients: Validation of a new self-reported questionnaire of adherence. *Photodermatology, Photoimmunology and Photomedicine*, *36*(2), 118–125. doi:10.1111/phpp.12519 31596975

[CIT0005] Clarke, A., Thompson, A. R., Jenkinson, E., Rumsey, N., & Newell, R. (2013). *CBT for appearance anxiety: Psychosocial interventions for anxiety due to visible difference*. Chichester: John Wiley & Sons, Ltd.

[CIT0006] Coulter, A., Entwistle, V. A., Eccles, A., Ryan, S., Shepperd, S., & Perera, R. (2015). Personalised care planning for adults with chronic or long-term health conditions. *Cochrane Database of Systematic Reviews*, *2012*(3), 1–129. doi:10.1002/14651858.CD010523.pub2 PMC6486144 25733495

[CIT0007] Dixon, D., & Johnston, M. (2010). Health behaviour change competency framework: Competences to deliver interventions to change lifestyle behaviours that affect health. *Scottish Government*. Retrieved from: http://www.healthscotland.com/documents/4877.aspx

[CIT0008] Durand, M.-A., Carpenter, L., Dolan, H., Bravo, P., Mann, M., Bunn, F., & Elwyn, G. (2014). Do interventions designed to support shared decision-making reduce health inequalities? A systematic review and meta-analysis. *PLoS ONE*, *9*(4), e94670. doi:10.1371/journal.pone.0094670 24736389 PMC3988077

[CIT0009] Easthall, C., & Barnett, N. (2017). Using theory to explore the determinants of medication adherence: Moving away from a one-size-fits-all approach. *Pharmacy*, *5*(3), 50–58. doi:10.3390/pharmacy5030050 PMC5622362 28970462

[CIT0010] Fassihi, H. (2013). Spotlight on ‘xeroderma pigmentosum’. *Photochemistry and Photobiology*, *12*, 7884. doi:10.1039/c2pp25267h 23132518

[CIT0011] Graham, E. (2016). *Improving outcomes through personalised medicine*. NHS England, Medical Directorate. Medicines, Diagnostics and Personalised Medicine Unit. Publications Gateway Reference: 05784.

[CIT0012] Hargraves, I., LeBlanc, A., Shah, N. D., & Montori, V. M. (2016). Shared decision making: The need for patient-clinician conversation, not just information. *Health Affairs*, *35*(4), 627–629. 27044962 10.1377/hlthaff.2015.1354

[CIT0013] Harris, R. (2013). *Getting unstuck in ACT: A clinician's guide to overcoming common obstacles in acceptance and Commitment Therapy*. Oakland, CA: New Harbinger Publications.

[CIT0014] Hawkins, R. P., Kreuter, M. W., Resnicow, K., Fishbein, M., & Dijkstra, A. (2008). Understanding tailoring in communicating about health. *Health Education Research*, *23*(3), 454–466. doi:10.1093/her/cyn004 18349033 PMC3171505

[CIT0015] Holmes, E. A., Hughes, D. A., & Morisson, V. L. (2014). Predicting adherence to medications using health psychology theories: A systematic review of 20 years of empirical research. *Value in Health*, *17*(8), 863–876. doi:10.1016/j.jval.2014.08.2671 25498782

[CIT0016] Johnstone, L., & Dallos, R. (2014). *Formulation in psychology and psychotherapy*. East Sussex: Routlegde.

[CIT0017] Krebs, P., Prochaska, J. O., & Rossi, J. S. (2010). Defining what works in tailoring: A meta-analysis of computer-tailored interventions for health behavior change. *Preventive Medicine*, *51*(3-4), 214–221. doi:10.1016/j.ypmed.2010.06.004 20558196 PMC2939185

[CIT0018] Kreuter, M., Strecher, V. J., & Glassman, B. (1999). One size does not fit all: The case for tailoring print materials. *Annals of Behavioral Medicine*, *21*(4), 276–283. doi:10.1007/BF02895958 10721433

[CIT0019] Kreuter, M., & Wray, R. J. (2003). Tailored and targeted health communication: Strategies for enhancing information relevance. *American Journal of Health Behavior*, *27*(Suppl 3.), S227–S232. doi:10.5993/AJHB.27.1.s3.6 14672383

[CIT0020] Kunneman, M., Montori, V., Castaneda-Guarderas, A., & Hess, E. P. (2016). What is shared decision making? And What is it not. *Academic Emergency Medicine*, *23*(12), 1320–1324. doi:10.1111/acem.13065 27770514

[CIT0021] Lehmann, A. R., McGibbon, D., & Stefanini, M. (2011). Xeroderma pigmentosum. *Orphanet Journal of Rare Diseases*, *6*, 70. doi:10.1186/1750-1172-6-70 22044607 PMC3221642

[CIT0022] Lustria, M. L. A., Noar, S. M., Cortese, J., Van Stee, S. K., Glueckauf, R. L., & Lee, J. (2013). A meta-analysis of web-delivered tailored health behavior change interventions. *Journal of Health Communication*, *18*(9), 1039–1069. doi:10.1080/10810730.2013.768727 23750972

[CIT0023] Michie, S., Richardson, M., Johnston, M., Abraham, C., Francis, J., Hardeman, W., … Wood, C. E. (2013). The Behavior change Technique Taxonomy (v1) of 93 hierarchically clustered techniques: Building an international consensus for the reporting of behavior change interventions. *Annals of Behavioral Medicine*, *46*(1), 81–95. doi:10.1007/s12160-013-9486-6 23512568

[CIT0024] Morgan, M., Anderson, R., Walburn, J., Weinman, J., & Sarkany, R. (2019). The influence of perceived medical risks and psychosocial concerns on photoprotection behaviours among adults with xeroderma pigmentosum: A qualitative interview study in the UK. *BMJ Open*, *9*, e024445. doi:10.1136/bmjopen-2018-024445 PMC6377541 30782905

[CIT0025] National Research Council. (2011). *Toward precision medicine: Building a knowledge network for biomedical research and a new taxonomy of disease*. Washington, DC: The National Academies Press. 22536618

[CIT0026] NHS England. (2018a). *Service specification: DNA nucleotide excision repair disorders service (all ages)*. Retrieved from https://www.england.nhs.uk/publication/service-specification-dna-nucleotide-excision-repair-disorders-service-all-ages/

[CIT0027] NHS England. (2018b). *Highly specialised services*. Retrieved from https://www.england.nhs.uk/commissioning/spec-services/highly-spec-services/

[CIT0028] Nikcevic, A. V., Kuczmierczyk, A. R., & Bruch, M. (2006). *Formulation and treatment in clinical health psychology*. East Sussex: Routlegde.

[CIT0029] Noar, S. M., Benac, C. N., & Harris, M. S. (2007). Does tailoring matter? Meta-analytic review of tailored print health behavior change interventions. *Psychological Bulletin*, *133*(4), 673–693. doi:10.1037/0033-2909.133.4.673 17592961

[CIT0030] Noar, S. M., Grant Harrington, N., Van Stee, S. K., & Shemanski Aldrich, R. (2011). Tailored health communication to change lifestyle behaviors. *American Journal of Lifestyle Medicine*, *5*(2), 112–122. doi:10.1177/1559827610387255

[CIT0031] Peters, G. J., Ruiter, R. A., & Kok, G. (2013). Threatening communication: A critical re-analysis and a revised meta-analytic test of fear appeal theory. *Health Psychology Review*, *7*(Suppl 1), S8–S31. doi:10.1080/17437199.2012.703527 23772231 PMC3678850

[CIT0032] Petrie, K. J., Cameron, L. D., Ellis, C. J., Buick, D., & Weinman, J. (2002). Changing illness perceptions after myocardial infarction: An early intervention randomised controlled trial. *Psychosomatic Medicine*, *64*(4), 580–586. doi:10.1097/00006842-200207000-00007 12140347

[CIT0033] Petrie, K. J., Perry, K., Broadbent, E., & Weinman, J. (2012). A text message programme designed to modify patients’ illness and treatment beliefs improves self-reported adherence to asthma preventer medication. *British Journal of Health Psychology*, *17*(1), 74–84. doi:10.1111/j.2044-8287.2011.02033.x 22107110

[CIT0034] Petty, R. E., & Cacippo, J. T. (1986). The elaboration likelihood model of persuasion. *Advances in Experimental Social Psychology*, *19*, 123–205. doi:10.1016/S0065-2601(08)60214-2

[CIT0035] Rollnick, S., & Miller, W. R. (1995). What is motivational interviewing? *Behavioural and Cognitive Psychotherapy*, *23*(4), 325–334. doi:10.1017/S135246580001643X 19364414

[CIT0036] Sainsbury, K., Vieira, R., Walburn, J., Sniehotta, F. F., Sarkany, R., Weinman, J., & Araujo-Soares, V. (2018b). Understanding and predicting a complex behaviour using n-of-1 methods: Photoprotection in xeroderma pigmentosum. *Health Psychology*, *37*(12), 1145–1158. doi:10.1037/hea0000673 30221970

[CIT0037] Sainsbury, K., Walburn, J., Araujo-Soares, V., & Weinman, J. (2018a). Challenges and proposed solutions for formative research to inform systematic intervention development in rare and unstudied conditions: The case example of xeroderma pigmentosum. *British Journal of Health Psychology*, *23*(2), 229–237. doi:10.1111/bjhp.12287 29265545 PMC5901402

[CIT0038] Shay, L. A., & Lafata, J. E. (2015). Where is the evidence? A systematic review of shared decision making and patient outcomes. *Medical Decision Making*, *35*(1), 114–131. doi:10.1177/0272989X14551638 25351843 PMC4270851

[CIT0039] Tamura, D., DiGiovanna, J. J., Khan, S. G., & Kraemer, K. H. (2014). Living with xeroderma pigmentosum: Comprehensive photoprotection for highly photosensitive patients. *Photodermatology, Photoimmunology and Photomedicine*, *30*(2-3), 146–152. doi:10.1111/phpp.12108 PMC6334764 24417420

[CIT0040] Vidall, C., Barlow, H., Crowe, M., Harrison, I., & Young, A. (2011). Clinical nurse specialists: Essential resource for an effective NHS. *British Journal of Nursing*, *20*(17), S23–S27. doi:10.12968/bjon.2011.20.Sup10.S23 22067534

[CIT0041] Walburn, J., Anderson, R., & Morgan, M. (2019c). Forms, interactions, and responses to social support: A qualitative study of support and adherence to photoprotection amongst patients with xeroderma pigmentosum. *British Journal of Health Psychology*, doi:10.1111/bjhp.12396 PMC7004138 31756279

[CIT0042] Walburn, J., Canfield, M., Norton, S., Sainsbury, K., Araujo-Soares, V., Foster, L., et al. (2019a). Psychological correlates of adherence to photoprotection in a rare disease: International survey of people with xeroderma pigmentosum. *British Journal of Health Psychology*, *24*(3), 668–686. doi:10.1111/bjhp.12375 31183946 PMC6772157

[CIT0043] Walburn, J., Norton, S., Sarkany, R., Sainsbury, K., Araujo-Soares, V., Morgan, M., et al. (2019b). Evaluation of a personalised adherence intervention to improve photoprotection in adults with xeroderma pigmentosum (XP): Protocol for the trial of XPAND. *BMJ Open*, *9*, e028577. doi:10.1136/bmjopen-2018-028577 PMC6661555 31320353

[CIT1001] Walburn, J., Sainsbury, K., Foster, L., Weinman, J., Morgan, M., Norton, S., Canfield, M., Chadwick, P., Sarkany, R., & Araujo-Soares, V. (2020). Why? What? How? Using an Intervention Mapping approach to develop a personalised intervention to improve adherence to photoprotection in patients with Xeroderma Pigmentosum. *Health Psychology and Behavioral Medicine*, *8*(1), 475–500. 10.1080/21642850.2020.1819287 PMC8114411 34040882

[CIT0044] Wanyonyi, K. L., Themessl-Huber, M., Humphris, G., & Freeman, R. (2011). A systematic review and meta-analysis of face-to-face communication of tailored health messages: Implications for practice. *Patient Education and Counseling*, *85*(3), 348–355. doi:10.1016/j.pec.2011.02.006 21397434

